# Minimally invasive laparoscopic and robot-assisted emergency treatment of strangulated giant hiatal hernias: report of five cases and literature review

**DOI:** 10.1186/s13017-020-00316-1

**Published:** 2020-06-01

**Authors:** Graziano Ceccarelli, Alessandro Pasculli, Walter Bugiantella, Michele De Rosa, Fausto Catena, Fabio Rondelli, Gianluca Costa, Aldo Rocca, Mattia Longaroni, Mario Testini

**Affiliations:** 1 General Surgery, “San Giovanni Battista” Hospital, USL Umbria 2, Via Massimo Arcamone 1, 06034 Foligno, Italy; 2 Unit of General Surgery “V. Bonomo”, Department of Biomedical Sciences and Human Oncology, University of Bari “A. Moro”, Polyclinic of Bari, Piazza Giulio Cesare 11, 70124 Bari, Italy; 3 Department of Emergency and Trauma Surgery, Parma University Hospital, Viale Antonio Gramsci 11, 43126 Parma, Italy; 4 Department of Medicine and Health Sciences “V. Tiberio”, University of Molise, Via Francesco de Sanctis 1, 86100 Campobasso, Italy

**Keywords:** Giant hiatal hernia, Paraesophageal hernia, Emergency surgery, Laparoscopy, Robotic surgery

## Abstract

**Background:**

Giant hiatal hernia (GHH) is a condition where one-third of the stomach migrates into the thorax. Nowadays, laparoscopic treatment gives excellent postoperative outcomes. Strangulated GHH is rare, and its emergent repair is associated with significant morbidity and mortality rates. We report a series of five cases of strangulated GHH treated by a minimally invasive laparoscopic and robot-assisted approach, together with a systematic review of the literature.

**Methods:**

During 10 years (December 2009–December 2019), 31 patients affected by GHH were treated by robot-assisted or conventional laparoscopic surgical approach. Among them, five cases were treated in an emergency setting. We performed a PubMed MEDLINE search about the minimally invasive emergent treatment of GHH, selecting 18 articles for review.

**Results:**

The five cases were male patients with a mean age of 70 ± 18 years. All patients referred to the emergency service complaining of severe abdominal and thoracic pain, nausea and vomiting. CT scan and endoscopy were the main diagnostic tools. All patients showed stable hemodynamic conditions so that they could undergo a minimally invasive attempt. The surgical approach was robotic-assisted in three patients (60%) and laparoscopic in two (40%). Patients reported no complications or recurrences.

**Conclusion:**

Reviewing current literature, no general recommendations are available about the emergent treatment of strangulated hiatal hernia. Acute mechanical outlet obstruction, ischemia of gastric wall or perforation and severe bleeding are the reasons for an emergent surgical indication. In stable conditions, a minimally invasive approach is often feasible. Moreover, the robot-assisted approach, allowing a stable 3D view and using articulated instruments, represents a reasonable option in challenging situations.

## Background

Paraesophageal hernias represent 5 to 10% of all hiatal hernias (HH), and their incidence is increasing mainly because of the ageing of the population. They are classified into four types [1]. In type I (sliding HH), there is a widening of the muscular hiatal tunnel and circumferential laxity of the phrenoesophageal membrane, allowing a portion of the gastric cardia to herniate upwards. In type II, the gastric fundus herniates into the thorax, but the gastroesophageal junction maintains its normal position. Type III is the association of type II with the migration of the gastroesophageal junction into the thorax (it represents the 90% of all paraesophageal hernias). Type IV is associated with the migration of other viscera through the hiatus. Giant hiatal hernia (GHH) is a condition in which one-third or more of the stomach migrates into the thorax [2]. Primary GHH can be asymptomatic or can lead to chronic low-grade symptoms, such as heartburn, abdominal pain, early satiety, dysphagia, chest discomfort and dyspnoea. If they are present, there is a clear indication for elective surgical treatment. Severe acute symptoms are very uncommon [3, 4].

Rarely, GHH may present as an acute emergency due to the intrathoracic twisting of the stomach followed by mechanic obstruction. Acute gastric outlet symptoms, haemorrhage (mucosal bleeding of the stomach), gastric ischemia and perforation, are severe sequelae of strangulation or upside-down stomach [5, 6]. All these complications represent real life-threatening conditions, with a high mortality rate (up to 30%) [7, 8]. Other intra-abdominal organs, such as colon, spleen and small intestine, can be involved in the herniation [6, 7].

Since its first description in 1992, laparoscopic approach to symptomatic patients with both HH and GHH has gained broad consent and is now considered the gold standard for elective surgery, given its low mortality and morbidity [2, 7, 9–11]. Moreover, it is considered safe also in an emergency setting [12], especially in clinically stable patients. When available, even the robot-assisted approach may represent a valid option, giving the well-known postoperative benefits of minimally invasive procedures [13, 14]. However, in the case of unfit or unstable patients, the open approach remains mandatory.

Surgical emergency treatment provides the reduction of the migrated stomach with the excision of the hernia sac. The hiatal defect closure (direct or with mesh) may be followed by an anti-reflux procedure (according to Toupet or Nissen) or gastropexy [2, 10, 15, 16]. Nevertheless, prosthetic mesh reinforcement is overall accepted since its introduction has reduced the risk of recurrences [15, 17, 18]. Particular care is mandatory during gastric wall handling. Necrotic gastric wall or perforation may require gastric resection so that a fundoplication should be avoided. Collis-Nissen procedure may be necessary due to the presence of oesophageal shortening [19–21].

Compared to elective surgery, emergency procedures are related to a higher risk of morbidity and mortality [22, 23]. No high-level evidence is available about emergency management of this rare condition.

In a recent multi-institutional broad database study from the USA, robotic-assisted repair accounted for the 6% of all emergent cases and 8% of all minimally invasive treated ones [14]. Our aim, along with the evaluation of the feasibility and safety of the robotic-assisted approach, was to infer suggestions from the current literature about the minimally invasive management of GHH in emergency.

## Case series

During 10 years (December 2009–December 2019), 31 patients underwent surgery for GHH at our centre. All patients were treated using a robot-assisted or conventional laparoscopic surgical approach; the choice of robotic technology depended on the device’s availability. Among them, five cases were treated in an emergency setting because of complicated GHH. No open surgery cases were recorded in the same period. Informed consent for the surgical procedure was always obtained before the treatment.

Table 1 sums up the characteristics of the patients: mean age was 70 ± 18 years (range 47–88), and all patients were male. Most of the patients (60%) had a history of known symptomatic HH; in 3 cases, there was a history of gastroesophageal reflux disease (GERD).

**Table 1:** Case series

***N***	Age (y), sex	Symptoms^**a**^, examination^**b**^, history	Comorbidities	BMI kg/m^2^	CT findings	Endoscopic findings	Waiting time from admission to surgery (days)	Approach of hiatoplasty	Fundoplication	Mesh	Gastropexy/gastrostomy	Operative time (min)	Complications	Hospital stay (days)	Follow-up (minimum 6 months)
1	70, M	Symptomatic GHH	None	25.3	GHH with air-fluid level	Gastric obstruction	0	Robotic assisted	Nissen	No	None	190	None	4	Uneventful
2^c^	47, M	Weight loss, asymptomatic GHH	Depression, obesity	37.5	GHH with air-fluid level	Gastric obstruction. Diffuse ischemia of gastric mucosa^d^	0	Robotic assisted^e^	No	No	Foley 18 in the gastric antrum	230	antrum stenosis	21	Mild reflux, Foley removed on PD 35, endoscopic balloon dilatation
3	86, M	Weight loss, GERD, symptomatic GHH	Hypertension, dyslipidemia	27.4	Gastric volvulus	Gastric obstruction	2^f^	Laparoscopic	Toupet	No	Gastrostomy tube	180	None	9	Gastrostomy tube removed on PD 25
4	88, M	Asthenia, GERD, asymptomatic GHH	Atrial fibrillation in OAT, hypertension, COPD	24.4	GHH with upside-down stomach	Gastric obstruction	3 (stop of OAT)	Robotic assisted	Toupet	BIO-A®^g^	Gastrostomy tube	190	None	7	Gastrostomy tube removed on PD 20
5	59, M	Coffee ground vomiting, GERD, symptomatic GHH	Obesity	32.6	GHH with air-fluid level and signs of gastric wall vascular insufficiency	Not performed	0	3D laparoscopic	Nissen	BIO-A®^g^	Gastropexy	170	None	5	Uneventful

*CT* computed tomography, *GHH* giant hiatal hernia, *MOF* multi organ failure, *ICU* intensive care unit, *GERD* gastroesophageal reflux disease, *PD* postoperative day, *OAT* oral anticoagulant therapy, *COPD* chronic obstructive pulmonary disease, *BMI* body mass index

^a^Abdominal and thoracic pain, nausea and vomiting were always present. All patients were stable at admission

^b^In all cases, there was no/incomplete nasogastric tube progression

^c^In this case, elevated white blood cells count and lactate lead to the need of ruling out ischemic heart disease

^d^5 postoperative days in ICU

^e^Intraoperative gastroscopy to check the mucosa of the distal stomach

^f^Attempt of conservative approach

^g^Gore & Associates Inc, Newark, DE, USA

All patients referred to the emergency service complaining of severe abdominal and thoracic pain, nausea and vomiting lasting from some hours (case 2) to 3 days (case 5). Case 5 presented with coffee-ground vomit. In all cases, the examination revealed diffuse abdominal tenderness and incomplete or impossible nasogastric tube progression. Laboratory tests were not specific; nevertheless, in case 3, the elevated white cells count and lactate level, led to the need for ruling out ischemic heart disease.

All patients underwent electrocardiogram, chest X-ray and contrast-enhanced computed tomography (CT) scan of the chest and abdomen. The most common CT scan finding was air-fluid level in the context of a GHH. Most of the cases (80%) underwent endoscopy before surgery, that always showed gastric obstruction, and in case 2 diffuse ischemia of the gastric mucosa. Most of the patients (60%) underwent immediate surgery, except for case 3, where there was a conservative attempt because of the age, and for case 4, in which surgery occurred after 3 days of OAT stop. All patients showed stable hemodynamic conditions so that they could undergo a minimally invasive attempt. The average waiting time from admission to surgery was 1 day (0–3).

**Fig. 1:**
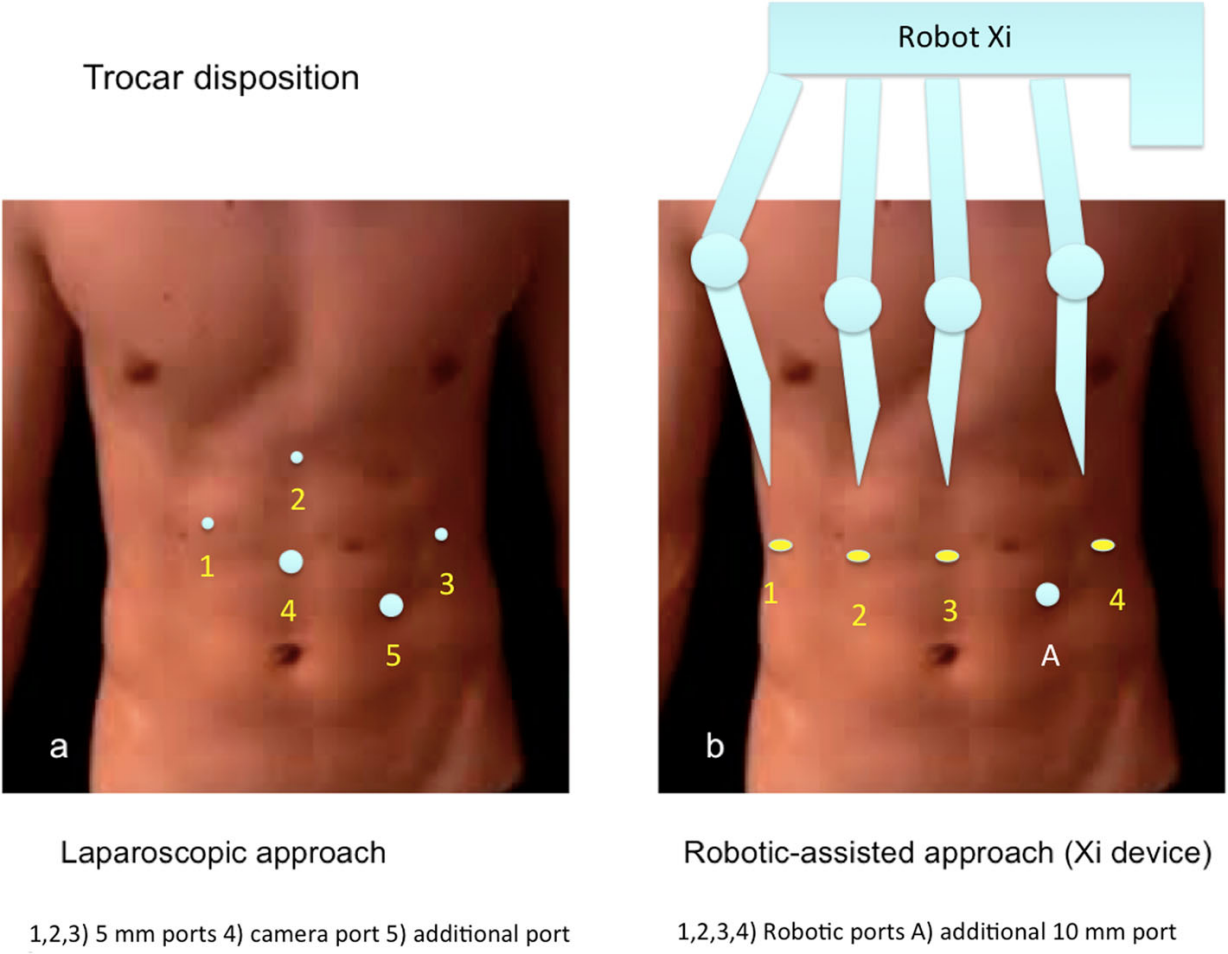
Trocar positions. **a** Laparoscopic approach. **b** Robotic-assisted approach

The surgical approach was robotic-assisted in 3 patients (60%), laparoscopic in the remaining 2. The Da Vinci Xi Robotic System (Intuitive Surgical, Inc. Sunnyvale, CA, USA) was used. The operation was always carried out by placing ports in the same fashion as an elective hiatoplasty (Fig. 1). The robotic cart was placed at the left side of the patient, at head-height. We placed the camera through the sopra-umbilical port, the monopolar cautery hook through the left one and the bipolar forceps through the middle-right port. The right robotic arm was used to retract the left liver lobe by the needle driver (replaced by another arm when needed for suturing). In robotically treated cases, we also placed an accessory 5-mm port. In laparoscopically treated cases, a 5-mm sub-xiphoidal port was used to retract the liver.

**Fig. 2:**
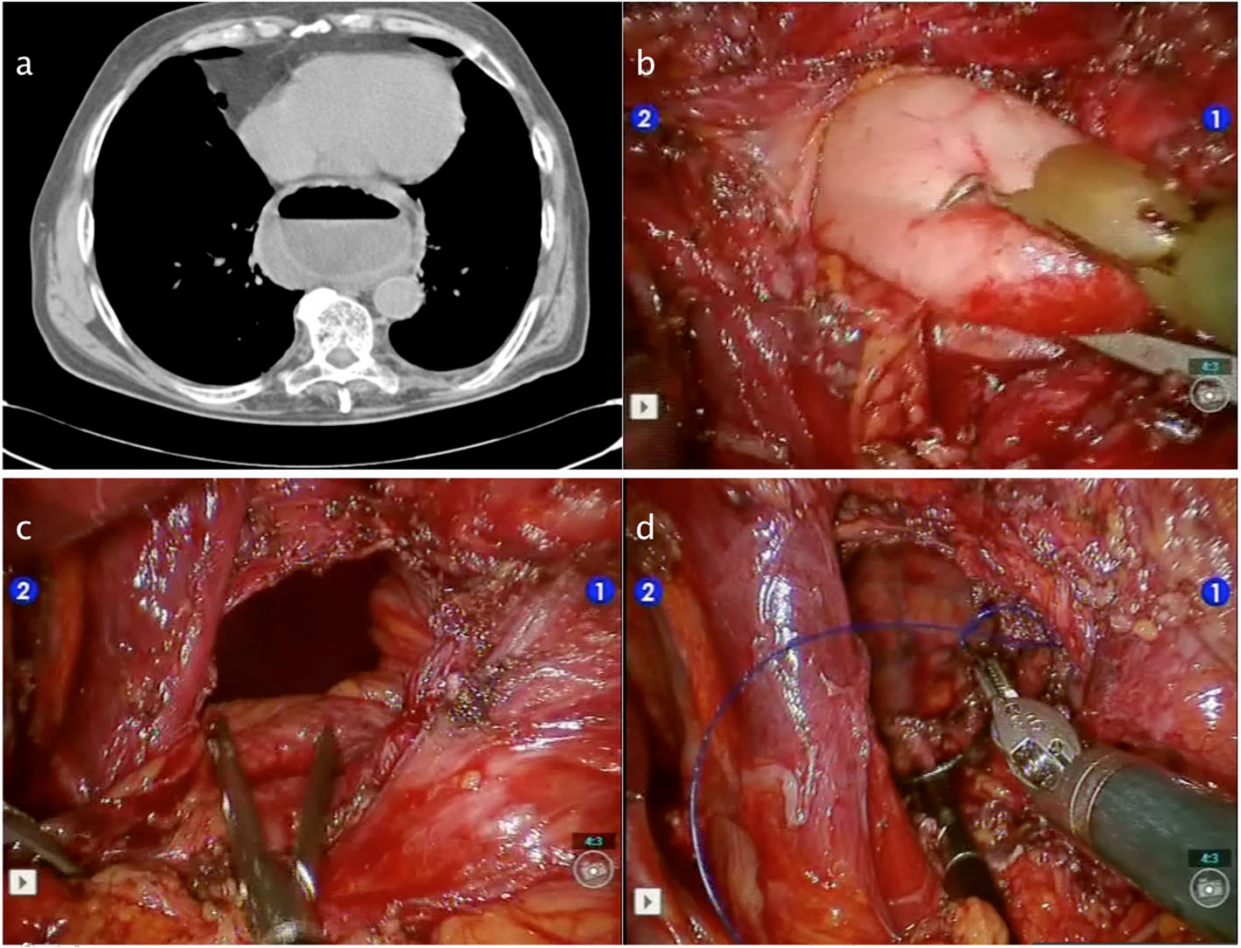
Case 1: Preoperative CT scan and intraoperative images. **a** CT scan showed a giant incarcerated hiatal hernia. **b** Intraoperative image of incarcerated hernia. **c** Hernia sac removed. **d** Crura closure

**Fig. 3:**
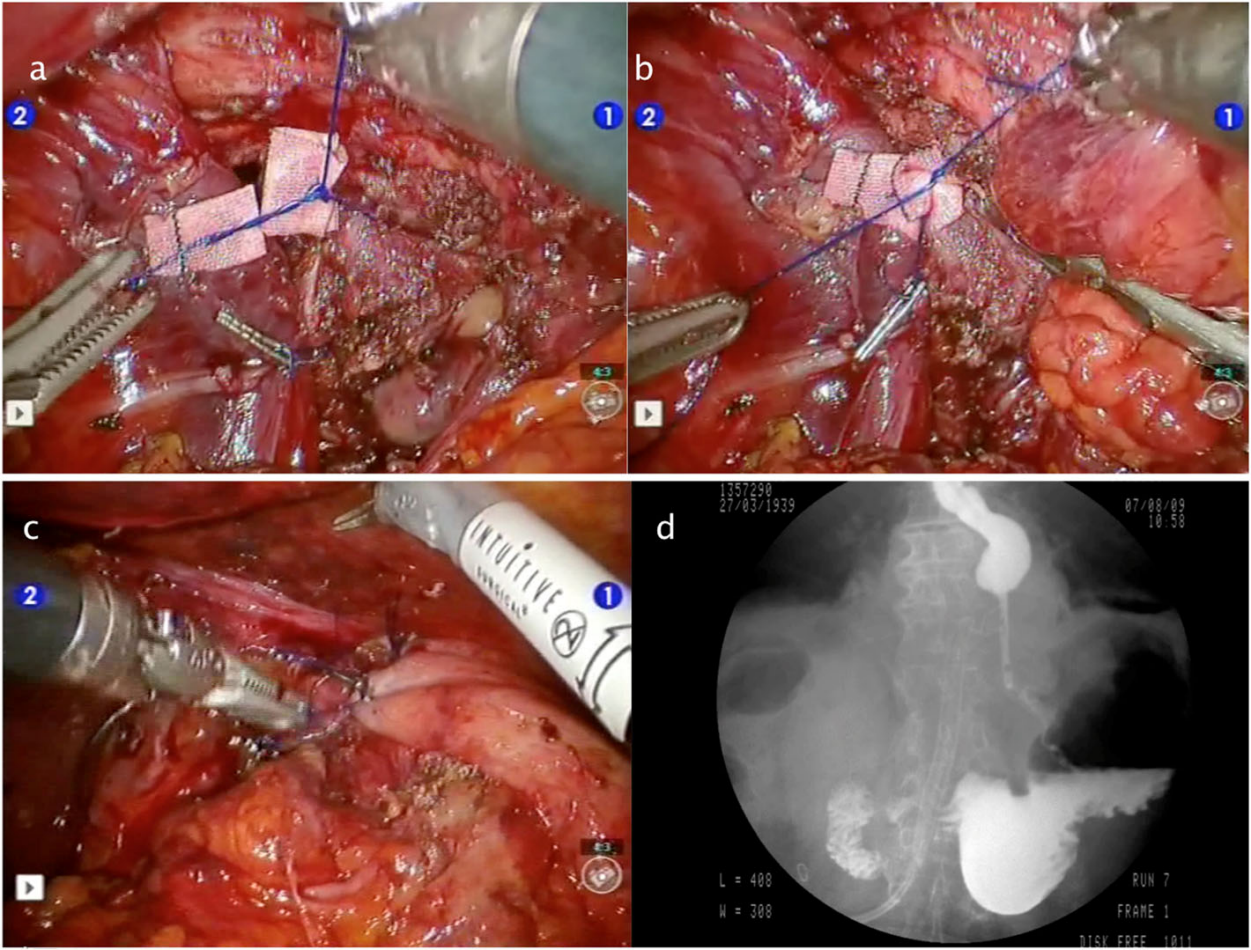
Case 1: Intraoperative images and postoperative X-ray. **a**–**b** Dacron pledgets reinforcement. **c** Nissen fundoplication. **d** X-ray showing a normal esophagogastric contrast transit

**Fig. 4:**
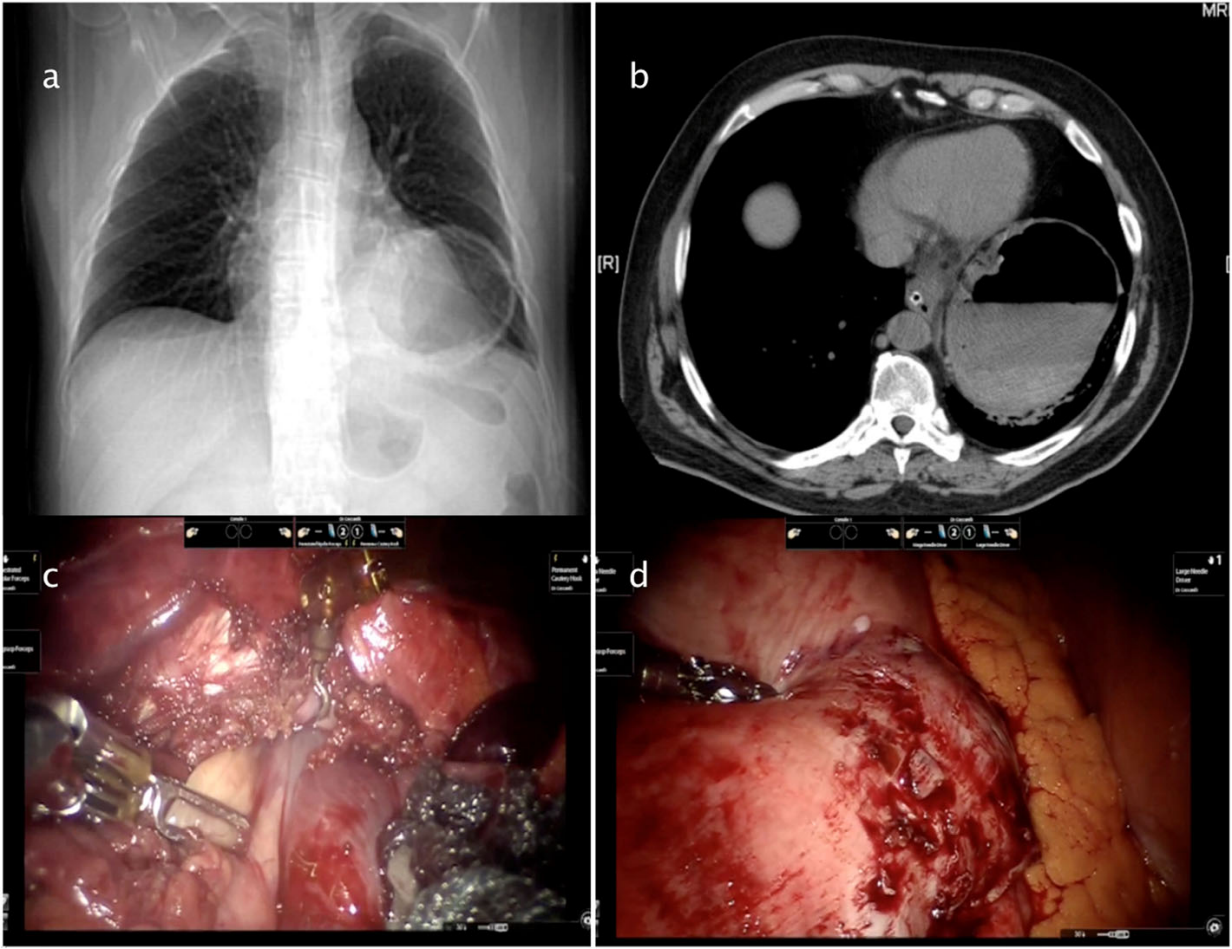
Case 2: Preoperative radiologic exams and intraoperative images. **a**–**b** Chest radiography and chest-abdomen CT scan showing an air-fluid level at the posterior mediastinum. **c**–**d** Gastric reduction after hiatal defect enlargement

**Fig. 5:**
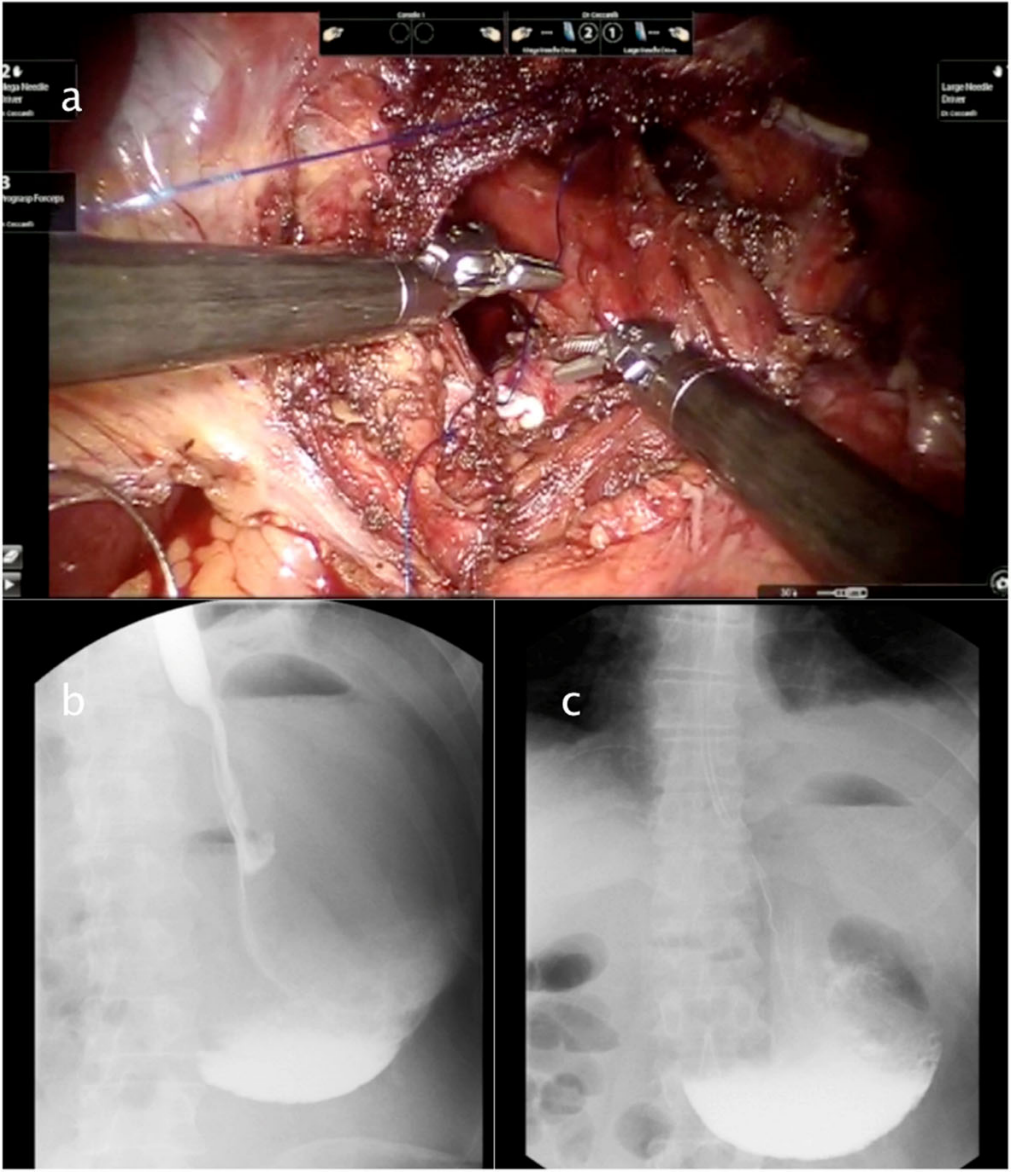
Case 2: Intraoperative images and postoperative X-ray. **a** Hiatoplasty. **b**–**c** A 10 postoperative days X-ray swallow showing a good oesophagogastric transit with antrum stenosis, treated by several pneumatic dilatation after patient discharge from the hospital

**Fig. 6:**
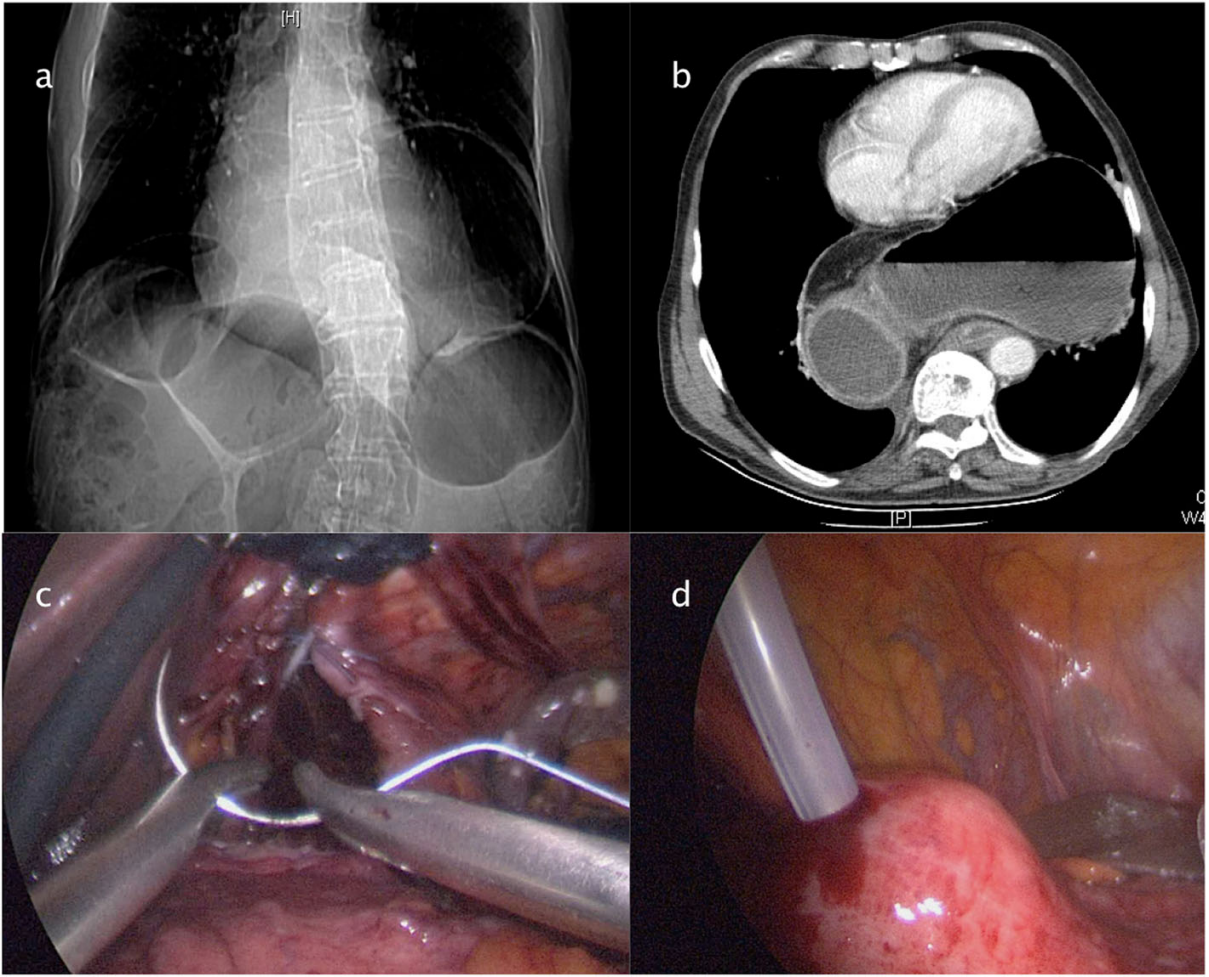
Case 3: Preoperative radiologic exams and endoscopic treatment. **a** Chest radiography showing migration of the stomach in the chest. **b** CT scan showing the stomach volvulus. **c** Crura closure by intracorporeal stitches. **d** Percutaneous endoscopic gastrostomy (PEG)

Figures 2, 3, 4, 5 and 6 reproduce imaging and intraoperative findings of the first three cases. The hook was used to lyse the adhesions of the hernia sac with mediastinal structures, while the herniated stomach and omentum were gently reduced in the abdomen. All patients showed type II/III hiatal hernias, with no cases of migration of other viscera through the hiatus. In case 2, the reduction of the hernia content in the abdomen was carried out only after the hiatal defect enlargement using ultrasound scalpel. After the reduction of the hernia content, the sac was removed, and the diaphragmatic crura and oesophagus were isolated. In the first 3 cases, the hiatoplasty was performed with polypropylene 2/0 stitches and non-absorbable pledgets. In the last 2 cases, a BIO-A (Gore & Associates Inc, Newark, DE, USA) resorbable prosthesis and pledgets were employed, secured with resorbable 2/0 stitches.

Four patients (80%) underwent Nissen and Toupet fundoplication: each in two cases, respectively. The choice between the two techniques depended on the obtainment of a floppy gastric valve. In case 2, because of the known ischemia of the gastric mucosa and the evidence of ischemic gastric serosa during the abdominal exploration, an intra-operative endoscopy was requested after the hernia reduction to check for the antrum and pylorus mucosa. After the careful evaluation of the whole stomach blood supply, we avoided gastric resection and fundoplication. In most of the cases (60%), a gastrostomy (cases 2, 3 and 4) secured the stomach to the abdominal wall. Case 5 underwent a gastropexy. The mean operative time was 192 ± 22.8 min.

The postoperative course was uneventful for most of the cases (80%). In case 2, the gastric mucosa ischemia and the difficult intubation led to a 5-day course in the intensive care unit, where the patient was monitored for the gastric perforation risk. The same patient developed antrum stenosis that healed after endoscopic treatment. The mean hospital stay was 9.2 ± 6.9 days (range 4–21).

Follow-up was carried out for a minimum of 6 months, with contrast X-ray of the upper gastrointestinal tract. Patients reported no complications or recurrences, and, when present, gastrostomy tubes were removed after 1 month from the operation.

## Review of literature

We performed a PubMed MEDLINE search during January 2020 using the following words: “hiatal hernia” AND “emergency OR emergent OR urgency OR urgent” AND “laparoscopy OR robotic OR minimally invasive”. Of the 77 found articles, we excluded articles not regarding humans (5), not in English (11) and not on adults (5). The 56 resulting papers were further investigated in terms of article design and subject matter excluding case reports and reviews (19) and articles about secondary hiatal hernias or elective procedures only (19); 18 articles were finally reviewed.

Table 2 shows data from the selected articles, reporting the number of patients not electively treated and through a minimally invasive approach. In those articles where it was not possible to obtain information regarding the urgent/emergent laparoscopically treated patients, we reported data from a larger cohort of patients, including those undergoing elective or open surgery.

**Table 2:** Review of literature

Article	Patients^**1**^	Age, sex^**2**^	Emergent indication	BMI^**3**^	ASA	Type of hernia	Use of mesh	Additional surgery	Hospital stay^**4**^	Main complications	Recurrences
Dubina [24]	266/3334	68 ± 14, 71% male	Obstruction or gangrene	n.a.	I–II (40%), III–IV (60%)^a^	n.a.	n.a.	n.a.	5 ± 7	Overall morbidity (11%), pneumonia (4%)^a^	n.a.
Shea [25]	30/229	74 ± 13, 80% male	Volvulus (47%), bleeding (17%), obstruction (20%), heart/respiratory failure (13%)	29 ± 6	II (23%), III (67%), IV (10%)	III (67%), IV (33%)	Acellular porcine liver derived (100%)	Nissen (100%), gastrostomy (13%), gastric resections (23%)	7 ± 6	Overall morbidity (47%)	17%
Zanotti [26]	1/4	73, female	Volvulus	n.a.	n.a.	III	Synthetic partially absorbable	Nissen	7	Pneumonia	No
Arevalo [27]	4/13	85 ± 9, sex n.a.	Bleeding (25%), obstruction (100%)	n.a.	III (50%), IV (50%)	n.a.	None	Gastropexy and gastrostomy (100%)	7 (2–14)	Reoperation for early recurrence (25%)	No
Augustin [28]	56/3498	71, 36% male	SIRS/sepsis/septic shock (30%)^b^	26 (25-28)^b^	III–IV (22%)^b^	n.a.	n.a.	n.a.	9 (7-10)^b^	Infections (7%), sepsis/shock (8%), reoperation (9%), heart/lung complications (28%), others (7%)^b^	n.a.
Light [29]	9/36	73^c^	Gastric volvulus (100%)^c^	n.a.	III–IV (73%)^c^	n.a.	None (no hiatal repair in 11%)^c^	Dor (22%), Nissen (8%), gastropexy (31%), gastric resections (11%)^c^	4	Pneumonia (25%), others (11%)^c^	2%
Klinginsmith [30]	1358/7950	65 ± 14, 48% male^d^	n.a.	n.a.	n.a.	n.a.	n.a.	Fundoplication (38%), gastrostomy (12%)	n.a.	Overall morbidity (8%)	n.a.
Köhler [31]	3/24	64 ± 14, 42% male^e^	Obstruction (100%)	n.a.	n.a.	n.a.	Synthetic (75%)^e^	Toupet (25%), Nissen (75%), gastropexy	n.a.	Pneumonia (33%), splenic bleeding (33%)	1%^e^
Jassim [32]	2896/41723	66, 35% male^f^	n.a.	Obesity (7%)^f^	n.a.	n.a.	n.a.	n.a.	n.a.	Overall morbidity (33%)^f^	n.a.
Mungo [33]	116/8186	63 ± 14, 28% male^g^	n.a.	< 25 (18%), 25–29.9 (27%), ≥ 30 (45%)^g^	II (56%), III (43%), IV (2%)^g^	n.a.	n.a.	n.a.	3 ± 5^g^	Overall morbidity (8%), reoperation (3%), pneumonia (2%), sepsis/shock (1%), heart/lung complications (4%)^g^	n.a.
Gebhart [34]	3/92	57 ± 14, 40% male ^h^	Incarceration	n.a.	n.a.	IV (2%)^h^	Biosynthetic (some cases)	Nissen (73%)^h^	2 ± 3^h^	Overall morbidity (6%)^h^	19%^h^
Parker [35]	25/266	75 (51–91), 12% male	Obstruction, haemorrhage, perforation, sepsis from gastric ischemia	30	n.a.	II (4%), III (92%), IV (4%)	Synthetic or acellular human dermal matrix (85%)	Nissen or Dor (76%), Collis (24%)	4 (1–13)	Overall morbidity (52%), heart/lung complications (24%), pneumonia (4%), infections (4%), leak (12%)	4%
Ballian [36]	24/980	< 50 (15%), 50–59 (12%), 60–69 (10%), 70–79 (17%), ≥ 80 (47%), 25% male^i^	n.a.	Underweight (44%), ideal (32%), overweight (17%), obese (15%), severely obese (15%)^i^	n.a.	n.a.	Unpecified (12%)^j^	Dor or Toupet (21%), Nissen (70%), Collis (47%)^j^	n.a.	Overall morbidity (23%), pneumonia (7%), heart/lung complication (18%), sepsis/shock (2%), leak (2%)^j^	1%^j^
Shaikh [37]	11/64	68 ± 2, 25% male	n.a.	n.a.	n.a.	n.a.	Porcine small intestine submucosa matrix used in 3 cases unspecified if emergent/elective, laparoscopic/open	Fundoplication (65%), gastropexy (13%), gastropexy only (25%)^k^	6 ± 2^†††^	Overall morbidity (23%)^k^	25%^k^
Louie [38]	9/58	78 (70–91), 41% male^l^	Incarceration (100%)	n.a.	n.a.	II (5%), III (78%), IV (17%)^l^	Biologic (38%)^‡^	Nissen (31%), Hill (33%), combined Nissen and Hill (34%), gastrostomy (2%)^l^	n.a.	Lung complications (11%)	11%
Bawahab [39]	17/20	71 (49–91), 15% male^m^	Obstruction, bleeding, respiratory failure	n.a.	n.a.	n.a.	Porcine small intestine submucosa matrix (6%)	Nissen or Dor (100%)	7 (2–15)	0%	n.a.
Parameswaran [40]	5/49	68 (38–90), 41% male^n^	n.a.	n.a.	III–IV (41%)^n^	II (10%), III (73%), IV (16%)^n^	PFTE or composite (22%), porcine small intestine submucosa matrix (12%)^n^	Nissen (100%), gastropexy in some cases^n^	n.a.	Overall morbidity (24%)^n^	8%^‡‡‡^
Hortsmann [41]	16	64 (36–80), 50% male	Obstruction	29 (19–31)	II (31%), III (69%)	n.a.	Polypropylene (100%)	Toupet (100%), gasropexy (100%)	9 (7–23)	Lung complications (31–38%)	0%

*BMI* body mass index, *ASA* American Society of Anaesthesiologists, *n.a.* not available, *SIRS* systemic inflammatory response syndrome, *PFTE* polytetrafluoroethylene

^a^Data available for the entire group of patients treated laparoscopically: 2473 patients, 266 treated in emergency setting

^b^Data available for the entire group of patients treated in emergency setting: 175 patients, 56 undergoing laparoscopic repair

^c^Data available for the entire group of patients treated for gastric volvulus: 36 patients, 9 undergoing laparoscopic repair

^d^Data available for the entire group of patients treated in emergency setting: 6726 patients, 1358 undergoing laparoscopic repair

^e^Data available for the entire group of studied patients

^f^Data available for the entire group of patients treated in emergency setting: 10,765 patients, 2896 undergoing laparoscopic repair

^g^Data available for the entire group of patients treated laparoscopically: 6415 patients, 116 treated in emergency setting

^h^Data available for the entire group of studied patients

^i^Data available for the entire group of patients treated non-electively: 199 patients, 24 undergoing laparoscopic repair

^j^Data available for the entire group of studied patients: 980 patients, 199 treated non-electively

^k^Data available for the entire group of patients treated laparoscopically: 40 patients, 11 treated in emergency setting

^l^Data available for the entire group of studied patients

^m^Data available for the entire group of studied patients

^n^Data available for the entire group of studied patients

^1^Number of patients treated in emergency setting and laparoscopically/total number of patients studied

^2^Mean ± standard deviation or median (range) for age expressed in years; percentage of male patients

^3^Mean ± standard deviation of BMI expressed in kg/m^2^

^4^Mean ± standard deviation or median (range) for hospital stay expressed in days

No randomised trial is available regarding the preoperative workup, nor the timing of surgery, nor the use and type of mesh or additional procedures, such as fundoplication, gastrostomy or gastropexy, so that no strong recommendations can be drawn.

Demographic data suggest no definite difference in sex distribution, although it seems that complicated hiatal hernia mostly affects the seventh and eighth decade [24–41]. The pathophysiology of HH is still unclear: increasing of intra-abdominal pressure and congenital or acquired widening of the diaphragmatic hiatus (weakness of the muscular crura due to elastin, collagens and matrix metalloproteinases abnormalities) are the most likely causes [42–44]. In the elderly, indeed, paraesophageal hernia is often asymptomatic, and the debate on whether to treat or not an asymptomatic patient is still active, following the high morbidity and mortality rate in elective repair [4, 10, 16]. Recently, pre-treatment patients’ characteristics were used to create a mortality and significant morbidity predictive model for GHH repair [16]. The 2013 Society of American Gastrointestinal and Endoscopic Surgeons (SAGES) guidelines recommend repair of all symptomatic paraesophageal hernias [20].

Literature suggests that patients with acute paraesophageal hernia are often overweight/obese (average body mass index from 29 to 30 kg/m^2^) [25, 35, 41] and with an American Society of Anaesthesiologists (ASA) score of III or higher (69–100%) [25, 27, 41].

Emergency surgery for incarcerated-strangulated GHH is mandatory especially in the presence of the classic Borchardt’s triad, consisting in retching, epigastric pain and failure to place a nasogastric tube. Generally, the patients present acutely with thoracic/abdominal pain and symptoms of mechanical gastric outlet obstruction (20–100%) [25, 27, 29, 31] or upper gastrointestinal bleeding (17–25%) [25, 27]. A chest X-ray should be the first diagnostic tool to rule out other diagnoses and to detect perforating signs [1, 20]. According to the selected articles, respiratory failure [25, 39] or even sepsis and shock [28, 35] are other possible acute presentations that appear to be the evolution of gastric ischemia. These unstable patients are often obliged to undergo immediate open surgery.

A chest-abdominal contrast-enhanced CT scan and upper endoscopy are the most important exams to be performed during the pre-operative workup. Endoscopy should be attempted with the aim of decompressing the stomach, having a direct view of mucosal condition (gastric ischemia), and trying a conservative management. Bleeding and gastric perforation, with septic sequelae, represent the clinical evolution if treatment is delayed.

There are no clear recommendations on the operative management of incarcerated hiatal hernia. Management algorithms for acutely presenting paraesophageal hernia were proposed on the results of a small series of patients [39]. The increased perioperative mortality and morbidity associated with the emergency repair for gastric volvulus and strangulation are reported in many series [22, 45]. Immediate open surgery is suggested in case of unstable patients. In other cases, an initial laparoscopic approach is advisable for trained surgical teams. Emergent laparoscopic HH reduction and repair may be carried out in cases without gastric perforation, with low morbidity rate and with the benefits of a minimally invasive approach [30, 35, 39]. The selected literature reported an average hospital stay of 4–9 days [25–27, 29, 35, 39, 41], overall morbidity rate of 0–52% [25, 30, 35] and a recurrence rate of 0–17% [25, 29, 35, 38, 41].

With a few exceptions [29], a hiatoplasty is always performed with different suture strategies (direct, pledgets and meshes). There is no evidence regarding the advisable use and type of mesh: both synthetic [26, 31, 35, 40, 41] and biologic mesh [25, 35, 37–39] have been adopted. About fundoplication, the main factors that influence the decision are gastric fundus tissue condition and the history of GERD. Nissen fundoplication is reported up to 75–100% of cases and is the most common technique [25, 31]. Additional techniques, such as gastrostomy, gastropexy, Collis procedure or gastric resections, are required in selected cases, and this underlines to what extent this surgery is tailored case by case.

Nevertheless, no previous article, among the selected ones, ever reported a predominant robotic approach for the emergent paraesophageal hernia repair. In our case series, age was not an exclusion criterion to minimally invasive approach. We believe that, in the acute setting, the concerns about the postoperative risk of recurrence are negligible compared to the risk of gastric perforation (after gastric vascular sufferance) and the absence of functional study (oesophageal manometry, swallow x-ray exam, etc.). The surgical goals, indeed, appear to be different case by case, and techniques should be carefully tailored.

This study has some limitations: the limited number of cases in our experience prevents from drawing any general conclusion; moreover, no open procedures were accounted both in emergency and in elective setting, so any comparison was not possible. Still, the selected literature shows the absence of comprehensive data about the outcome after emergent robotic-assisted repair of GHH.

## Conclusions

The robotic-assisted approach in acute paraesophageal hernia repair may give advantages compared to conventional laparoscopy, for what concerns the surgeon’s comfort and precision, and this is probably unique in emergency surgery. However, its use mainly depends on device availability and the surgical team’s experience.

We can conclude, on the basis of current literature, that it is challenging to stigmatise recommendations for the management of GHH in an emergency setting. In many cases, a minimally invasive approach is possible and safe, with good peri- and postoperative outcomes. Robotic surgery may represent an appealing option to ease some surgical steps as dissection, abdominal reduction and suturing.

## Data Availability

All data generated or analysed during this study are included in this published article.

## Abbreviations

GHH: Giant hiatal hernia
CT: Computed tomography
HH: Hiatal hernia
GERD: Gastroesophageal reflux disease
OAT: Oral anticoagulant therapy
SAGES: Society of American Gastrointestinal and Endoscopic Surgeons
ASA: American Society of Anaesthesiologists
